# Characteristics Associated with Choosing Long-Acting Reversible Contraception in Rural Guatemala: A Secondary Analysis of a Cluster-Randomized Trial

**DOI:** 10.26502/ogr062

**Published:** 2021-06-25

**Authors:** Margo S Harrison, Saskia Bunge-Montes, Claudia Rivera, Andrea Jimenez-Zambrano, Gretchen Heinrichs, Antonio Bolanos, Edwin Asturias, Stephen Berman, Jeanelle Sheeder

**Affiliations:** 1 University of Colorado, Anschutz Medical Campus, Aurora, USA; 2 Fundación para la Salud Integral de los Guatemaltecos, Quetzaltenango, Guatemala; 3 Denver Health, Denver, USA

**Keywords:** Postpartum Contraception, LARC, SARC, Implant, Guatemala

## Abstract

**Design:**

We conducted a secondary analysis of a cluster-randomized trial to observe characteristics associated with women who chose to use long-acting reversible contraceptives (LARC) compared to those who chose a short-acting method 12 months after enrollment.

**Methods:**

The trial studied four control and four intervention clusters where the intervention clusters were offered contraception at their 40-day routine postpartum visit; control clusters received standard care, which included comprehensive postpartum contraceptive counseling. Women were followed through twelve months postpartum.

**Results:**

The study enrolled 208 women; 94 (87.0%) were in the intervention group and 91 (91.0%) were in the control group. At twelve months, with 130 (70.3%) women using contraception at that time. 94 women (50.8%) were using a short acting method compared to 33 (17.9%) who chose a long-acting method, irrespective of cluster. In mixed effect regression modeling adjusted for cluster, characteristics associated with a reduced likelihood of choosing long-acting contraception in multivariate modeling included age (aRR 0.98 [0.96,0.99], p = 0.008) and any education (compared to no education; aRR 0.76 [0.60,0.95], p = 0.02). Women who were sexually active by their enrollment visit (40 days postpartum) were 30% more likely to opt for a long-acting method (aRR 1.30 [1.03,1.63], p = 0.03).

**Conclusion:**

Older and more educated women were less likely to be using LARC a year after enrollment, while women with a history of early postpartum sexual activity were more likely to choose LARC.

## 1. Introduction

The World Health Organization supports provision of long-acting reversible contraceptives (LARC) in the postpartum setting to properly space and prevent undesired pregnancies because they have high satisfaction and continuation rates [1, 2]. The Pan-American Health Organization has advised that increased provision of LARC could achieve family planning goals for the Latin American region and should be promoted [3]. Use of postpartum LARC in Guatemala is moderate overall, but rates have historically been low in the Southwest Trifinio region [4-6]. Barriers to LARC have prevented expected utilization in this rural, agricultural community; in response to this gap in modern contraceptive provision, we implemented an intervention aimed to increased uptake of postpartum LARC by offering the implant and other contraceptives to women in their homes, free of charge, at their postpartum visits, in the setting of a cluster-randomized trial [5, 7]. We had a positive trial and found that uptake of the implant was higher in intervention than control clusters (25% vs 3%, p < 0.001) [4].

For this secondary analysis, our objective was to observe what characteristics were associated with women who chose LARC (the implant and the intrauterine device [IUD]) as compared to women who chose a shorter-acting option. Therefore, among women who were using contraception at the 12-month follow-up timepoint, we analyzed how those who chose a long-acting compared to those who chose a short-acting method (SARC). For the purposes of this analysis, LARC included the implant and the intrauterine device (although only the implant was included as part of the study intervention), and SARC included contraceptive pills, condoms, and the injection.

## 2. Methods

### 2.1 Design

This study is a secondary analysis of a prospective, non-blinded, cluster-randomized trial; the protocol has been published for further detail on the original trial [5].

### 2.2 Setting

In Southwest Guatemala there is region known locally as the Southwest Trifinio where the University of Colorado has a collaboration with a local agribusiness [8]. Together, the organizations established the Center for Human Development, which is a local clinic that also serves as an umbrella organization for community-based maternal and child health nursing programming [8]. The maternal program provides antepartum and postpartum care in the home setting and is called Madres Sanas [8]. The cluster-randomized trial was layered on top of this healthcare infrastructure [5].

### 2.3 Population

**Figure 1:**
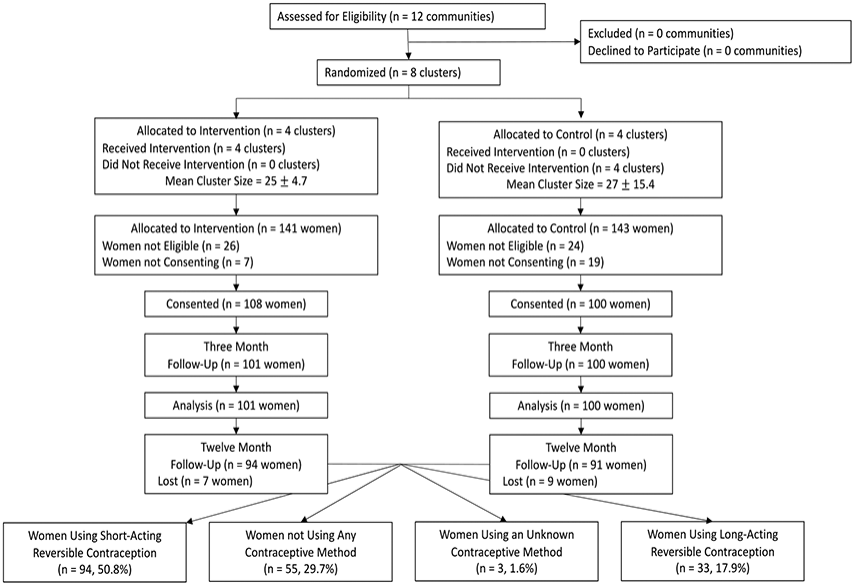
Consort diagram.

For the purposes of this secondary analysis, all women who were followed through 12 months post-enrollment without missing data on whether or not they were using contraception at that time were considered in this analysis. Women were excluded if they were not using a contraceptive method, or there was missing data on the type of contraceptive method they were currently using. The population is shown in detail in Figure 1.

### 2.4 Outcomes

Our intention was, among the entire population of women who were using contraception by 12 months after enrollment in the study, to determine characteristics associated with women who chose LARC as compared to those using SARC.

### 2.5 Statistical methods

Descriptive statistics were used to generate percentages and counts of characteristics (sociodemographics, medical and obstetrical history) of the women using contraceptives overall and by type of contraceptive—LARC versus SARC. We performed comparisons of these characteristics in a mixed effects regression adjusted for cluster. All characteristics with a p-value < 0.20 were included in a multivariable model (mixed effects regression adjusted for cluster) to observe which were associated with LARC use 12 months post-enrollment. STATA software version 15.2 (StataCorp LP, College Station, TX, USA) was used for analysis.

## 3. Results

The flow diagram (Figure 1) presents the original cluster randomized trial with the populations for the current analysis listed at the bottom of the figure. By 12 months, 94 women from the intervention clusters and 91 from the control clusters were available for follow-up, which represents 88.9% of the originally enrolled cohort. 94 (50.8%) of the cohort was using SARC by 12 months, 55 (29.7%) were not using a method, 3 (1.6%) were missing data on the type of contraception used, and 33 (17.9%) were using LARC.

Table 1 describes the 127 women who were using contraception by 12 months post-enrollment in terms of their contraceptive choice. No women were using condoms, 4 (3.2%) were using the contraceptive pill, 90 (70.8%) were using the injectable contraceptive, 28 (22.0%) had an implant in situ, 1 (0.8%) had obtained an IUD, and 4 (3.2%) women in the cohort had been surgically sterilized. Table 2 describes the subpopulations of women using SARC and LARC as well as the overall cohort. The women in the study had a median age of 23 years old, with most having had some education (around 90%), and they were predominantly not single (90%). The cohort had a large primiparous subpopulation (35%) and most women had been visited at least four times in the course of their pregnancy (80%) by the Madres Sanas nurses. Almost two-thirds (60%) of participants had a vaginal birth with a skilled birth attendant, with 46% delivering in a facility setting, and almost three-fourths birthed an infant weighing 2500 grams or more. By the 72-hour postpartum visit most babies were still alive (92%) and almost half were female. By the 40-day postpartum visit, 9.5% of women had been sexually active and over one-third of women reported not desiring future fertility (36%).

Women choosing LARC as compared to those choosing SARC were younger (median around 20 years versus 24), p < 0.05. The comparison groups were otherwise similar in bivariate comparisons on education (82% with some education versus 92%), marital status (85% married versus 92%), 33% primiparous versus 35%, and received four or more Madres Sanas visits (70% versus 84%), p > 0.05. The groups had a similar prevalence of vaginal birth (52% versus 63%), delivering at the hospital (39% versus 48%), delivery by a traditional birth attendant (28% versus 34%), of having an infant 2500 grams or more (70% versus 80%), and having that infant be male (45.5% versus 41.5%), p > 0.05. Most babies in both groups were born alive (82% versus 96%) and women reporting not desiring future fertility in 30% versus 38% of the cohorts, respectively, p > 0.05. Postpartum sexual activity was borderline different between the groups with 73% of women choosing LARC being inactive and 90% of women choosing SARC being inactive, p = 0.047. The primary outcome is presented in Table 3, which compared characteristics associated with LARC utilization by 12 months post-enrollment as compared to women who were using SARC at the time of analysis. All covariates with a p-value < 0.20 were included in the regression, which included maternal age, maternal education, and sexually activity by 40 days postpartum. As women aged, they were slightly less likely to choose LARC by 12 months post-enrollment (aRR 0.98 [0.96,0.99], p = 0.008). If women had any education, they were also less likely to choose LARC than more educated women (aRR 0.76 [0.60,0.95], p = 0.02). With respect to sexual activity by 40 days postpartum, women who were sexually active by that timepoint were 30% more likely to be using LARC 12 months later (aRR 1.30 [1.03,1.63], p = 0.03).

## 4. Discussion

Our cluster-randomized parallel-arm pragmatic trial designed to test the hypothesis that reducing barriers to accessing contraception would increase implant usage at 3 months, was a successful trial. This analysis shows that by 12 months, 70.3% of the population was using contraception, and 22.3% of that usage was attributable to LARC devices. The only characteristic associated with an increase use of LARC at that timepoint was having been sexually active in the early postpartum period, with older age and having some level of education reducing that likelihood. Regarding the finding of increasing age associated with a slightly reduced likelihood of using LARC, we believe this finding is due to a very high utilization of female sterilization as a means of contraception in this community [4]. Eligibility criteria excluded women over age 35 and those who were already using a contraceptive method [5]. Less than 10 women were excluded for age and almost 50 were excluded because they had already been sterilized. We believe given the historically high rates of sterilization as a means of controlling undesired fertility in this community and the high rates of women who had to be excluded for already having been sterilized, it is likely that this very common method of contraception accounts for the reduced likelihood of opting for LARC as women age [5]. We hypothesize that instead of choosing LARC, women are choosing sterilization, and in fact in one of our other analyses, we found that the only women switching away from the implant did so in favor of tubal ligation (data under review). We know that sterilization is one of the more common methods of contraception in Latin America and globally in low-resource settings [3, 9].

Our finding regarding education is slightly less clear to us and might benefit from more categorizations to further delineate the findings. We can only surmise that this finding is consistent with our conjecture about our age finding; we propose that more educated women may choose the most definitive method of controlling fertility (sterilization) over a reversible contraceptive device. Education has been shown to be associated with a greater likelihood of modern contraceptive use, but our finding regarding education and reduced LARC utilization should be confirmed, as prior research has suggested that increased education is associated with increased LARC uptake [10, 11]. We were pleased to note the finding that women who have already had intercourse by 40 days postpartum were 30% more likely to choose LARC. We could consider conducting an analysis of our population, generally, to determine what is associated with early sexual activity and ensure the implant is available for that population as we know those women are likely to opt for it, in an effort to prevent short interval pregnancy. This would be an interesting and easy area for quality improvement work through the Madres Sanas program. We believe that our trial was very successful as having 70% of women using contraception 12 months postpartum is high, as is the rate of LARC utilization at over one in five women [7]. Our trial, because it was pragmatic, may have resulted in varied counseling by nurse team regarding contraceptives, which may have in turn been associated with variable use in the postpartum setting that could affect the results we have presented. Additionally, this was a secondary analysis and as such the data was not designed to answer this question, specifically. Our sample size, however, is relatively large as we had good follow-up rates, and the primary study question was related to postpartum contraceptive use, so there are strengths to the results, as well.

In conclusion, in this analysis we found that there was high use of LARC 12 months postpartum in the overall cohort and high use of the these effective methods in women participating in early postpartum sexual activity, which might be considered a high-risk group for short-interval pregnancy. We intend to build on this work by exploring how we can ensure LARC is available to this subset of the population through a quality improvement project.

## Figures and Tables

**Table 1:** Final method used by women followed through 12-months with a known contraceptive method.

Method	n (%) N = 127
Pills	4 (3.2)
Injection	90 (70.8)
Implant	28 (22.0)
Intrauterine Device	1 (0.8)
Female Sterilization	4 (3.2)

**Table 2:** Bivariate comparisons of women using short-acting verses long-acting reversible contraceptives.

	Total Population (n = 127)	Short Acting (n = 94, 74.0%)	Long Acting (n = 33, 26.0%)	P-Value
**Sociodemographic Characteristics**				
Age in years (median IQR)	23.2 [19.0,25.6]	23.8 [19.6,26.4]	20.4 [17.5,24.3]	**0.02**
Education				**0.09**
None	13 (10.2%)	7 (7.5%)	6 (18.2%)	
Any	113 (89.0%)	86 (91.5%)	27 (81.8%)	
Missing	1 (0.8%)	1 (1.0%)	0 (0.0%)	
Married				0.50
Yes	114 (89.8%)	86 (91.5%)	28 (84.8%)	
No	11 (8.7%)	7 (7.5%)	4 (12.2%)	
Missing	2 (1.6%)	1 (1.0%)	1 (3.0%)	
**Obstetric and Antepartum Characteristics**				
Parity				0.59
0	1 (0.8%)	0 (0.0%)	1 (3.0%)	
1	44 (34.6%)	33 (35.1%)	11 (33.3%)	
2	35 (27.5%)	25 (26.6%)	10 (30.3%)	
3	20 (15.8%)	16 (17.0%)	4 (12.1%)	
4+	27 (21.3%)	20 (21.3%)	7 (21.2%)	
Number of Madres Sanas Prenatal Visits				0.92
<4	16 (12.6%)	12 (12.8%)	4 (12.1%)	
4+	102 (80.3%)	79 (84.0%)	23 (69.7%)	
Missing	9 (7.1%)	3 (3.2%)	6 (18.2%)	
**Delivery Characteristics**				
Mode of Delivery				0.80
Vaginal Birth	76 (59.8%)	59 (62.8%)	17 (51.5%)	
Cesarean Birth	43 (33.9%)	32 (34.0%)	11 (33.3%)	
Missing	8 (6.3%)	3 (3.2%)	5 (15.2%)	
Location of Delivery				0.95
Home, Private Clinic, or Other	60 (47.2%)	46 (48.9%)	14 (42.4%)	
Facility (Hospital)	58 (45.7%)	45 (47.9%)	13 (39.4%)	
Missing	9 (7.1%)	3 (3.2%)	6 (18.2%)	
Birth Attendant				0.63
Comadrona (TBA, “unskilled”)	41 (32.3%)	32 (34.0%)	9 (27.8%)	
Nurse or Physician (“skilled”)	78 (61.4%)	59 (62.8%)	19 (57.6%)	
Missing or “I don’t know”	8 (6.3%)	3 (3.2%)	5 (15.2%)	
Birthweight at Delivery				0.36
≤ 2500g	14 (11.0%)	9 (9.6%)	5 (15.2%)	
2500g+	98 (77.2%)	75 (79.8%)	23 (69.7%)	
Missing	15 (11.8%)	10 (10.6%)	5 (15.2%)	
**Postpartum Characteristics**				
Sex of Infant				0.49
Male	54 (42.5%)	39 (41.5%)	15 (45.5%)	
Female	63 (49.6%)	50 (53.2%)	13 (39.4%)	
Missing	10 (7.9%)	5 (5.3%)	5 (15.2%)	
Infant Outcome				0.14
Bom Alive, died before 72-hour visit	1 (0.8%)	0 (0.0%)	1 (3.0%)	
Bom Alive, alive at 72-hour visit	117 (92.1%)	90 (95.7%)	27 (81.8%)	
Missing	9 (7.1%)	4 (4.3%)	5 (15.2%)	
Sexual Activity Since Birth				0.047
Yes	12 (9.5%)	6 (6.4%)	6 (6.2%)	
No	109 (85.8%)	85 (90.4%)	24 (72.7%)	
Missing	6 (4.7%)	3 (3.2%)	3 (9.1%)	
Desired Timeframe Until Next Pregnancy				0.98
Approximately 2 years	1 (0.8%)	1 (1.0%)	0 (0.0%)	
Approximately 3 years	4 (3.2%)	4 (4.3%)	0 (0.0%)	
> 3 years	44 (34.6%)	30(31.9%)	14 (42.4%)	
I don’t know	27 (21.3%)	20 (21.3%)	7 (21.2%)	
No more children desired	46 (36.2%)	36 (38.3%)	10 (30.3%)	
Missing	5 (3.9%)	3 (3.2%)	2 (6.1%)	

Note: p-values represent a linear model adjusted for cluster

**Table 3:** Multivariable Model of Characteristics Associated with Use of a Long-Acting Postpartum Contraceptive Method as Compared to a Short Acting Method.

Characteristic	aRR	Cl	P-Value
Age (continuous)	0.98	0.96,0.99	0.008
Any Education (reference: No Education)	0.76	0.60,0.95	0.02
Sexual Activity by 40 Days Postpartum (reference: No Sexual Activity)	1.30	1.03,1.63	0.03

All characteristics with p < 0.01 in bivariate comparisons included in multivariable model (linear model adjusted for cluster); this included: maternal age, maternal education, and sexual activity before 40 days postpartum

## Abbreviations:

WHO: World Health Organization
PAHO: Pan American Health Organization
LARC: Long-acting reversible contraceptives
SARC: Short-acting reversible contraceptives
